# Vδ2 T cells are associated with favorable clinical outcomes in patients with bladder cancer and their tumor reactivity can be boosted by BCG and zoledronate treatments

**DOI:** 10.1136/jitc-2022-004880

**Published:** 2022-08-24

**Authors:** Sylvain Nguyen, Mathieu F Chevalier, Sulayman Benmerzoug, Valérie Cesson, Anna K Schneider, Sonia-Cristina Rodrigues-Dias, Florence Dartiguenave, Ilaria Lucca, Patrice Jichlinski, Beat Roth, Denise Nardelli-Haefliger, Laurent Derré

**Affiliations:** 1 Urology Research Unit and Urology Biobank, Department of Urology, Centre Hospitalier Universitaire Vaudois, Lausanne, Switzerland; 2 INSERM U976, HIPI Unit (Human Immunology, Pathophysiology and Immunotherapy), Hôpital Saint-Louis, Paris, France

**Keywords:** Urinary Bladder Neoplasms, Immunity, Immunotherapy

## Abstract

**Background** Bladder cancer is an important public health concern due to its prevalence, high risk of recurrence and associated cost of management. Although BCG instillation for urothelial cancer treatment is the gold-standard treatment for this indication, repeated BCG treatments are associated with significant toxicity and failure, underlining the necessity for alternative or complementary immunotherapy and overall for better understanding of T-cell responses generated within bladder mucosa. Tumor-infiltrating lymphocytes (TIL) have long been recognized as a crucial component of the tumor microenvironment for the control of tumor. Among TIL, unconventional γδ T cells sparked interest due to their potent antitumor functions. Although preclinical mouse xenograft models demonstrated the relevance of using γδ T cells as a novel therapy for bladder cancer (BCa), the contribution of γδ T cells in BCa patients’ pathology remains unaddressed.

**Methods** Therefore, we first determined the proportion of intratumor γδ T cells in muscle-invasive patients with BCa by deconvoluting data from The Cancer Genome Atlas (TCGA) and the frequency of blood Vδ1, Vδ2, and total γδ T cells, by flow cytometry, from 80 patients with BCa (40 non-muscle and 40 muscle-invasive patients with BCa), as well as from 20 age-matched non-tumor patients. Then we investigated in vitro which treatment may promote BCa tumor cell recognition by γδ T cells.

**Results** We observed a decrease of γδ T-cell abundance in the tumor compared with corresponding normal adjacent tissue, suggesting that the tumor microenvironment may alter γδ T cells. Yet, high intratumor γδ T-cell proportions were significantly associated with better patient survival outcomes, potentially due to Vδ2 T cells. In the blood of patients with BCa, we observed a lower frequency of total γδ, Vδ1, and Vδ2 T cells compared with non-tumor patients, similarly to the TCGA analysis. In addition, a favorable clinical outcome is associated with a high frequency of circulating γδ T cells, which might be mainly attributed to the Vδ2 T-cell subset. Furthermore, in vitro assays revealed that either BCG, Zoledronate, or anti-BTN3 agonistic antibody treatment of bladder tumor cells induced Vδ2 T-cell cytolytic (CD107a^+^) and cytokine-production (IFN-γ and TNF-α). Strikingly, combining BCG and Zoledronate treatments significantly elicited the most quantitative and qualitative response by increasing the frequency and the polyfunctionality of bladder tumor-reactive Vδ2 T cells.

**Conclusions** Overall, our results suggest that (1) Vδ2 T cells might play a prominent role in bladder tumor control and (2) non-muscle invasive patients with BCa undergoing BCG therapy may benefit from Zoledronate administration by boosting Vδ2 T cells’ antitumor activity.

**What is already known on this topic**

- γδ T cells have a potent antitumor activity, which led to the development of new γδ T cell-based therapies for different types of cancer. However, the clinical significance of γδ T cells in bladder cancer has not been elucidated yet.

**What this study adds**

- Intratumor and circulating Vδ2 T cells are associated with good clinical outcomes. Their functional properties can be boosted by Zoledronate and BCG treatment of bladder tumor cell lines.

**How this study might affect research, practice or policy**

- Vδ2 T cells may play a prominent role in bladder tumor control and might be a new prognostic biomarker. By enhancing Vδ2 T-cells antitumor functions, combining Zoledronate with BCG may be a promising treatment in non-muscle invasive patients with bladder cancer.

## Introduction

Bladder cancer (BCa) is a highly prevalent disease associated with a substantial propensity for tumor recurrence.^1^ Even though BCG therapy is the gold-standard treatment for non-muscle-invasive bladder cancer (NMIBC) patients, this treatment is limited by significant side effects and a high failure rate, since 40%–60% of patients experience recurrence 5 years after treatment. This underlines the urgent need for novel therapies.^2 3^ Nowadays, it is well established that tumor-infiltrating lymphocytes (TIL) are crucial in the immune surveillance of several cancer types, including BCa.^4 5^ However, prior studies have mainly focused on conventional αβ T cells, while unconventional γδ T cells remain largely understudied. In humans, γδ T cells encompass two major subsets, harboring either a Vδ1 or Vγ9Vδ2 (hereafter called Vδ2) T-cell receptor (TCR). Vδ1 T cells preferentially reside in the epithelial compartment, while Vδ2 T cells constitute the main peripheral blood γδ T-cell subset.^6^ Nonetheless, it has been observed that Vδ2 T-cell infiltration can occur in various human solid tumors.^7 8^ Both γδ T-cell subsets display potent cytotoxic capacity and recognize distinct tumor antigen moieties.^9^ For instance, Vδ1 T cells can recognize MHC-like molecules, such as CD1c, CD1d, or MICA/B.^9^ However, the relevance in vivo of all these Vδ1 TCR ligands are still debated. In contrast, Vδ2 T cells sense non-peptidic phosphorylated antigens (PAgs) that are overproduced by transformed cells, such as the isopentenyl pyrophosphate (IPP), through the conformation change of butyrophilin 3A1 (BTN3A1) molecules.^10^ Tumor cells recognition by Vδ2 T cells can be easily manipulated in vitro by using agonistic α-BTN3 antibodies (eg, Clone: 20.1)^11 12^ or by pharmacologically increasing the intracellular level of IPP using amino bisphosphonates (eg, Zoledronate).^13^ Interestingly, it has been shown that PAgs isolated from BCG extract potentiate Vδ2 T-cell proliferation and cytotoxicity.^14^ Furthermore, intravesical administration of γδ T cells and Zoledronate (Zol) significantly reduced tumor growth in an orthotopic xenograft mouse model of BCa.^15^ More recently, it has been shown in mice that rapamycin improves γδ T-cell activity thus sharpening BCG-mediated antitumor immunity.^16^ Given that murine and human γδ T cells do not share obvious homologies between TCR genes, and mouse γδ T cells do not respond to phosphoantigens, interpretations of such study in mice are limited, highlighting the need of further investigations in humans. To date, almost no data are available on the frequency and the function of γδ T cells in patients with BCa. Therefore, this study aims to determine the clinical relevance of γδ T cells in patients with BCa and investigate how different treatment could improve their function against bladder tumor cells.

## Material and methods

### Study populations and patient-derived biological materials

Forty NMIBC and 40 MIBC patients were recruited at the Lausanne University Hospital. Peripheral blood from NMIBC and MIBC patients was collected the day before the transurethral resection of the bladder tumor (TURBT) or cystectomy, respectively. Twenty age-matched (median: 69 years, IRQ: 61.5–76) non-tumor patients with mainly acute and chronic bladder inflammation or cystitis and no previous history of BCa, were enrolled. Because of hematuria and BCa suspicion, they all underwent a TURBT, which was ultimately negative. Moreover, blood samples from healthy donors (HD) were collected from the local Swiss blood bank. Peripheral blood monocytic cells (PBMC) from HD and patients were isolated by Isopaque-Ficoll density gradient centrifugation and immediately cryopreserved in RPMI supplemented with 40% FCS and 10% DMSO. For NMIBC patients undergoing a BCG therapy, urine samples were obtained after each instillation to expand urinary T cells, as described elsewhere.^17^ Patients’ characteristics are described in online supplemental table 1–3.

10.1136/jitc-2022-004880.supp2 Supplementary data

### Computational analysis

Gene expression data for bladder urothelial cancer (UCa) from TCGA^18^ were obtained with corresponding clinical information UCSC Xena (https://xenabrowser.net). TCGA dataset includes 405 samples from patients with localized and locally advanced BCa (T2 to T4), as well as 19 non-tumor paired adjacent tissue samples. Expression values of mRNA were transformed as X=log2(X+1), where X represents RNA Seq V2 RSEM values. Then, in order to obtain the relative frequency of total γδ T cells infiltrating advanced bladder tumors, this TCGA dataset was entered into Immune Cell Abundance Identifier (ImmuCellAI; http://bioinfo.life.hust.edu.cn/ImmuCellAI%23!/), a gene set signature-based algorithm able to deconvolute the relative abundance of different immune cell subsets from bulk transcriptome dataset.^19^ The following gene list was used for the determination of γδ T-cell abundance in ImmuCellAI: *KLRG1*, *CYP4A11*, *CCR5*, *GZMH*, *ACD*, *CHST12*, *GZMA*, *GZMB*, *LAG3*, *NKG7*, *PRF1*, *PVRIG*, *TINF2*, *ZMAT5*, *C1orf61*, *GNLY*, *LCP2*, *PSTPIP1*, *PTPN4*, *RALY*, TAB2, and *TDP1*.^19^ For the calculation of *TRDC*/*TRGC1*/*TRGC2* (as a surrogate for pan-γδ T-cell signature), *TRDC*/*TRGC1* (Vδ2 T-cell signature), and *TRDC*/*TRGC2* (non-Vδ2 T-cell signature) gene signature score, patient with undetectable TRDC, or TRGC1/TRGC2 gene expression (log2(normalized mRNA value +1)≤0) were filtered, and the mean value of each gene in the gene set was calculated, as described elsewhere.^20–22^

### Flow cytometry analysis

The following monoclonal antibodies (mAb) were used at predetermined optimal concentrations: α-CD3-PC7 (UCHT1), α-CD4-BV711 (OKT4), α-CD45-BV650 (HI30), and α-TCRVδ2-AF700 (B6, Biolegend); α-CD8-BUV395 (G42-8), and α-panTCRγδ-PE (11F2, BD Biosciences); α-TCRVδ1-FITC (TS8.2, Thermo Fisher Scientific). For cell surface antigens staining, cells were incubated with FcR Blocking Reagent (Miltenyi) to block unspecific binding, stained with mentioned mAb for 20 min at 4°C, and subsequently labeled with amine-reactive dye (Aqua LIVE/DEAD Stain Kit, Thermo Fisher Scientific) to allow dead cells exclusion. For the evaluation of BTN3 expression on tumor cell lines, cells were labeled with α-BTN3 (Clone: 20.1, mouse IgG1, Invitrogen) as described above, washed, then subsequently stained with polyclonal antibody α-mouse IgG-PE (eBioscience). For intracellular cytokine labeling, cells were fixed and stained for 30 min at room temperature using Intracellular Fixation and Permeabilization Buffer Set (eBioscience), α-IFN-γ-BV421 (4S.B3), and α-TNF-α-AF647 (Mab11, Biolegend). Stained cells were acquired on either Gallios (Beckman Coulter) or LSRFortessa (BD Biosciences), and data were analyzed with FlowJo (TreeStar) and SPICE V.6.1.

### Evaluation of Vδ2 T-cell cytotoxic capacities and cytokine production

Human UCa cell lines Bu68.08 (NMIBC; G2; Ta)^23^ and J82 (MIBC; T3)^24^ were maintained in RPMI supplemented with 10% FCS. Cell lines were infected with OncoTICE BCG (MSD) at a multiplicity of infection of 10 for 16 hours, and/or treated with Zol (Zometa, Novartis) at 5 µM for 12 hours, and/or with an agonistic α-BTN3 antibody specific for A1, A2, and A3 isoform (Clone: 20.1, mouse IgG1, Invitrogen) at 10 µg/mL for 2 hours. As negative control, cell lines were treated at 10 µg/mL of mouse IgG1 κ isotype control (MOPC-21, BD Biosciences) with the same regimen used for α-BTN3 antibody treatment. In the case of combinatorial treatments, treatments were applied successively. Then cells were washed three times with PBS to remove any residual treatment. Finally, treated cell lines were cocultured with PBMCs from patients with HD and BCa or urinary T-cell lines for 6 hours at effector/target ratio of 2:1. Protein Transport Inhibitor Cocktail (eBioscience) and α-CD107a-BV605 (H4A3, Biolegend) were added in culture media at the initiation of the coculture assay. Staining for cell surface markers and intracellular cytokines were carried out as described above.

### Statistical analysis

All data are represented as mean±SE of the mean. The difference between means was assessed by t-test for data with normal distribution and Mann-Whitney non-parametric test for non-normal variables. In online supplemental figure 4D, Wilcoxon test was performed. For multiple comparisons, a one-way analysis of variance followed by post-hoc Dunnet’s test was performed. For Kaplan-Meier analysis, cutoffs (c/o) were determined with ‘OptimalCutpoints’ R package using the criteria minimizing the Euclidian distance between the ROC plot and the reference point (0,1).^25 26^ Survival curve comparisons were assessed by log-rank test. Comparison of γδ T-cell polyfunctionality was carried out by partial permutation test. All statistical analyses were carried out by Graphpad PRISM 9, except for the polyfunctionality permutation test, which was performed on SPICE V.6.1.

10.1136/jitc-2022-004880.supp1 Supplementary data

## Results

### γδ T-cell intratumoral abundance and BTN3A1 gene expression correlate with improved survival in patients with BCa

**Figure 1:**
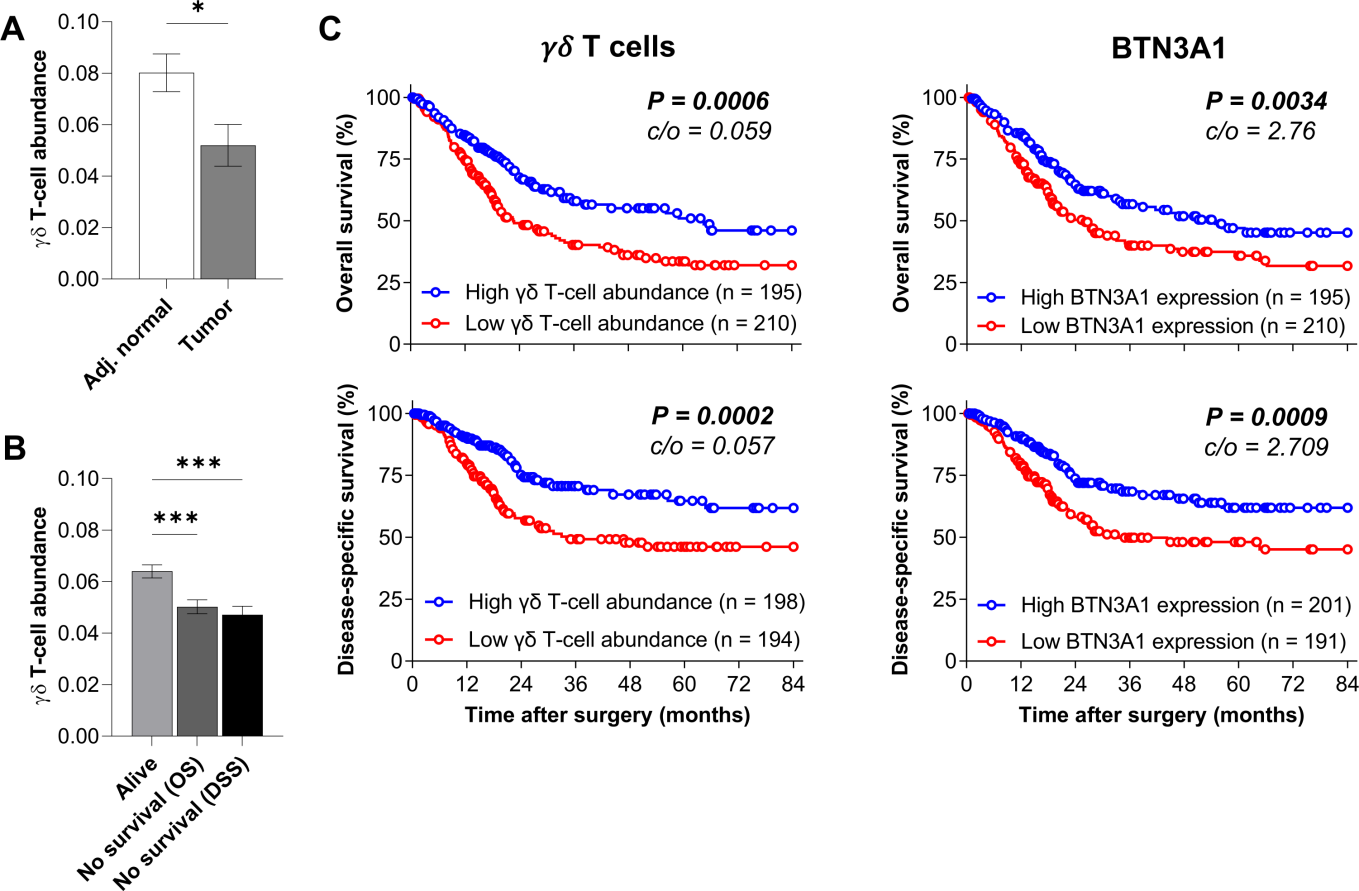
Intratumor γδ T-cell abundance and BTN3A1 expression in MIBC patients from the TCGA cohort. (A) γδ T-cell abundance in bladder tumor tissue and paired adjacent normal tissue from MIBC patients from the TCGA dataset (n=19). (B) Intratumor γδ T-cell abundance in MIBC patients from the TCGA cohort according to their survival status. Indicated *P* values were determined by one-way ANOVA. Overall (n=405) and disease-specific (n=392) survival analysis based on γδ T-cell abundance (C) and *BTN3A1* mRNA expression (D) obtained from TCGA cohort. *P<0.05. ***p<0.001. ANOVA, analysis of variance; MIBC, muscle-invasive bladder cancer; TCGA, The Cancer Genome Atlas.

As the contribution of γδ T cells in BCa patients’ pathology remains unknown, we first performed a large-scale analysis of MIBC data from TCGA. Transcriptomic data were subjected to the ImmuCellAI algorithm, a gene set signature-based method, allowing the deconvolution of the immune cell subset abundance.^19^ We observed that γδ T-cell abundance was diminished in the tumor site compared with corresponding normal adjacent tissue (figure 1A). Interestingly, we found that patients, who did not survive during the clinical follow-up exhibited less intratumoral γδ T-cell abundance than patients who did survive (figure 1B). Next, survival analyses showed that patients with a high γδ T-cell abundance had significant improved overall and disease-specific survival (figure 1C), suggesting that γδ T cells may be involved in bladder tumor control. Then, we assessed whether the proportion of RNA transcripts coding for butyrophilin subfamily 3 (BTN3) members could correlate with patients’ survival. We observed that high BTN3A1, but also BTN3A2, and BTN3A3, expression correlates with better survival (figure 1D and online supplemental figure 1). Since BTN3A1 is a critical activating ligand for Vδ2 T cells,^12^ this result suggests that Vδ2 T-cell subsets might be involved in bladder tumor control. Owing to the significant overlap of γδ T-cell transcriptional signatures with those of other immune cell subsets, especially NK and CD8 T cells,^22 27^ γδ T-cell abundance from bulk transcriptional data remains challenging to delineate accurately. Therefore, to validate results obtained with ImmuCellAI deconvolution, we performed survival analyses using γδ TCR gene signature (*TRDC, TRGC1, TRGC2*).^20 22^ As shown in online supplemental figure 2, patients with BCa with a high *TRDC/TRGC1/TRGC2* score (as a surrogate for pan γδ T-cell signature) or *TRDC/TRGC1* score (Vδ2 T-cell signature) show improved overall survival, while no difference was observed with *TRDC/TRGC2* score (non-δ2 T-cell signature). Overall, these results confirm that γδ T cells and potentially Vδ2 T cells might be involved in bladder tumor control.

### Circulating Vδ2 T cells correlate with improved survival in patients with BCa

**Figure 2:**
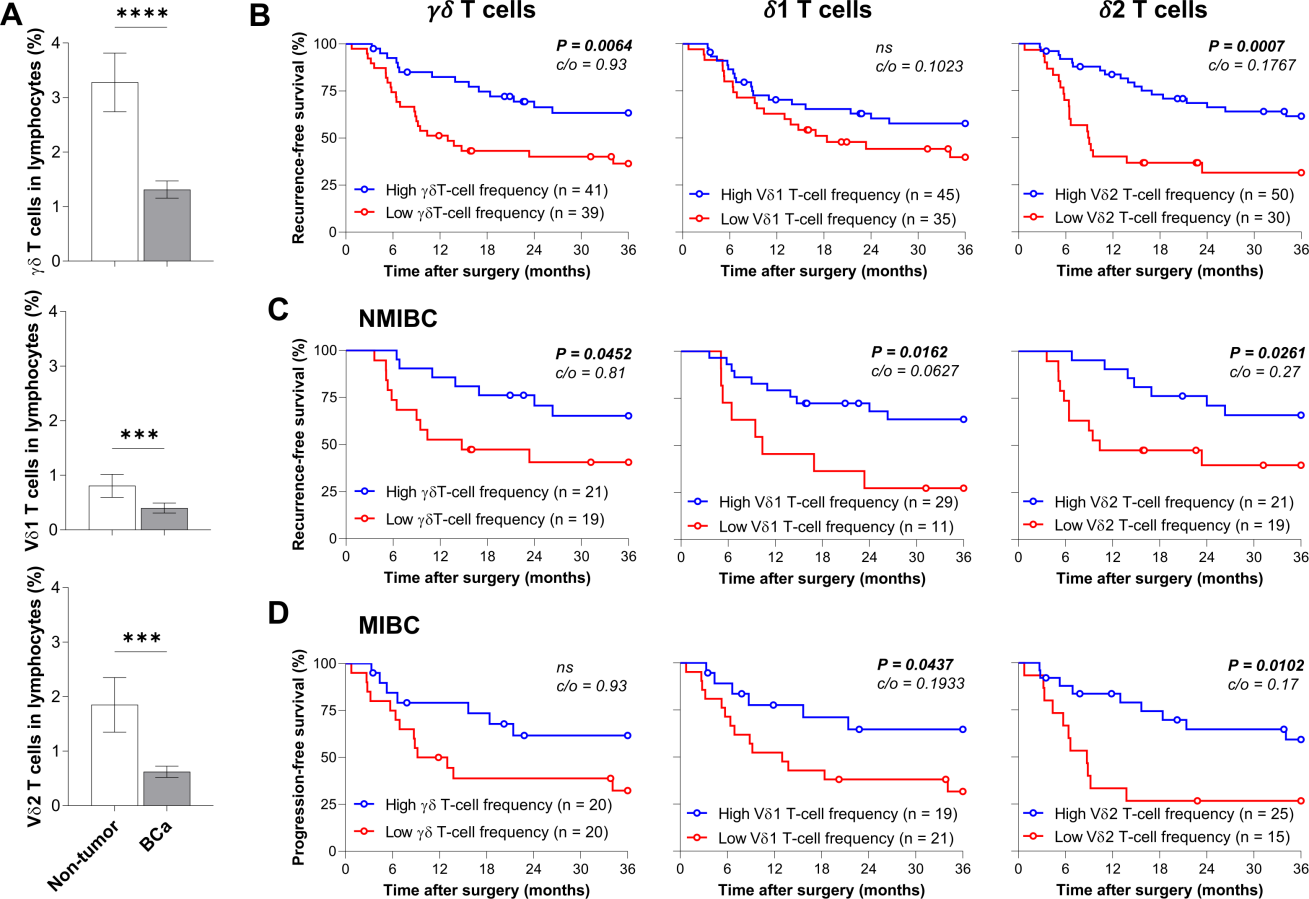
High frequency of peripheral blood Vδ2 T cells associates with low tumor recurrence and progression rates. (A) Frequency of circulating total γδ, Vδ1, and Vδ2 T cells in non-tumor (n=20) and patients with BCa (n=80) among live lymphocytes. Recurrence-free and progression-free survival of (B) BCa (n=80), (C) NMIBC (n=40), and (D) MIBC patients (n=40), based on their level of peripheral blood total γδ, Vδ1 or Vδ2 T cells. Cut-off values (c/o) are indicated. ***P<0.01. ****p<0.0001. BCa, bladder cancer; NMIBC, non-muscle-invasive bladder cancer.

To validate our observations, we determined by flow cytometry the frequency of circulating γδ T cells in a cohort of 80 patients with BCa, including 40 NMIBC and 40 MIBC patients, and in 20 age-matched non-tumor patients, as control. Of note, γδ T-cell population was identified as CD3^+^CD4^-^panTCRγδ^+^, while Vδ1 and Vδ2 T lymphocytes were defined as CD3^+^CD4^-^TCRVδ1^+^ and CD3^+^CD4^-^TCRVδ2^+^ subsets, respectively (online supplemental figure 3A). Similarly to the TCGA analysis, we observed a substantial reduction (>50%) of circulating total γδ, Vδ1 and Vδ2 T-cell proportions in patients with BCa compared with non-tumor age-matched patients (figure 2A), but no difference between NMIBC and MIBC patients (online supplemental figure 3B–D). Notably, although no difference was detected between tumor stages (data not shown), a lower frequency of blood Vδ2 T cell was found in NMIBC patients with high-grade tumor compared with NMIBC patients with low-grade tumor, suggesting that the bladder tumor microenvironment may alter γδ T cells (online supplemental figure 3E). Then, we performed survival analyses segregating our cohort according to the frequency of circulating γδ T cells or its subpopulations. Interestingly, patients with BCa with high γδ T-cell proportion had better recurrence-free survival (median was 13 months, while it was not reached in patients with high γδ T-cell frequency), which was mainly attributed to the Vδ2 T-cell subset (median was 9 months in patients with low Vδ2 T-cell frequency, while it was not reached in patients with high Vδ2 T-cell frequency) and not to Vδ1 T-cell frequency (figure 2B). When stratifying our cohort according to the bladder tumor type, although high γδ T cells are associated with a better survival only in NMIBC patients, high frequencies of both Vδ1 and Vδ2 T cells correlated with improved survival of NMIBC and MIBC patients (figure 2C, D). Overall, these data substantiate the role of γδ and Vδ2 T cells in anti-bladder tumor immune response.

### BCG, Zol, and agonistic α-BTN3 mAb promote Vδ2 T-cell antitumoral functions against BCa cell lines

**Figure 3:**
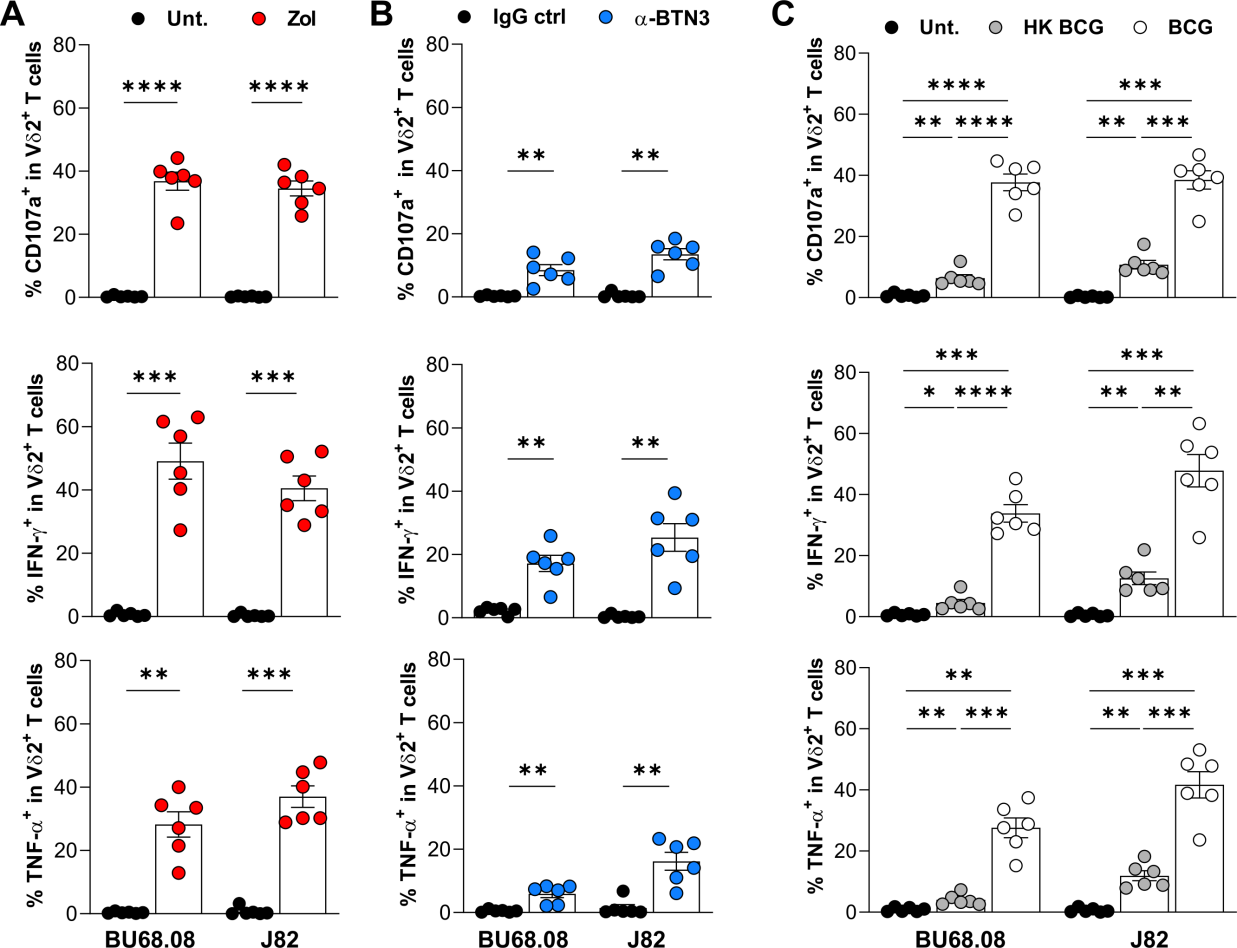
Vδ2 T-cell functional properties on stimulation with Zol-, agonistic α-BTN3 antibody-treated, or BCG-infected BCa cell lines. Measurement of CD107a, IFN-γ and TNF-α expression in Vδ2 T cells from PBMCs of HD (n=6) on coculture with BCa cell lines (BU68.08 and J82) treated by (A) Zol, (B) agonistic α-BTN3 antibody, or (C) BCG (MOI=10). *P<0.05. **p<0.01, ***p<0.001. ****p<0.0001. BCa, bladder cancer; HD, healthy donors; PBMCs, peripheral blood monocytic cells.

Since for both transcriptomic and flow cytometry analysis showed that Vδ2 T cells correlate with favorable BCa patients’ clinical outcomes, we investigated whether a treatment could promote BCa tumor cell recognition by Vδ2 T cells. Therefore, Bu68.08 and J82 cell lines, arising from non-muscle and muscle-invasive tumors, respectively, were treated with Zol (a drug promoting intracellular IPP accumulation), an agonistic α-BTN3 (Clone: 20.1) antibody, or with BCG. BCa cell lines were subsequently cocultured with PBMCs from HD, and Vδ2 T-cell cytokine production (IFN-γ and TNF-α) and cytotoxic potential (CD107a) were assessed by flow cytometry. Vδ2 T cells require the presence of BTN3A1 on tumor cells to detect intracellular IPP accumulation. Labeling bladder cancer cell lines with anti-BTN3 antibody (clone 20.1) confirmed the expression of BTN3 isoforms on bladder tumor cell lines treated or not (online supplemental figure 4A). As expected, Zol-induced IPP accumulation in both BCa cell lines promoted a robust cytotoxic response, as measured by the increase of the degranulation marker CD107a frequency, as well as a potent induction of IFN-γ- and TNF-α-producing Vδ2 T cells (figure 3A). Next, targeting the BTN3A1 receptor with an agonistic antibody also triggered Vδ2 T-cell functions, although with about twofold less extent than Zol treatment (figure 3B). On BCG infection, we observed a potent functional capacity, similarly to Zol treatment (figure 3C). Interestingly, Vδ2 T-cell functions were much less potently induced when BCa cell lines were treated with heat-inactivated BCG, suggesting that active BCG infection is required for optimal Vδ2 T-cell activation. In contrast, we did not observe any Vδ1 T-cell functional response against BCG-infected cell lines (online supplemental figure 4B).

### Additional effect of Zol and BCG on Vδ2 T-cell antitumoral functions

**Figure 4:**
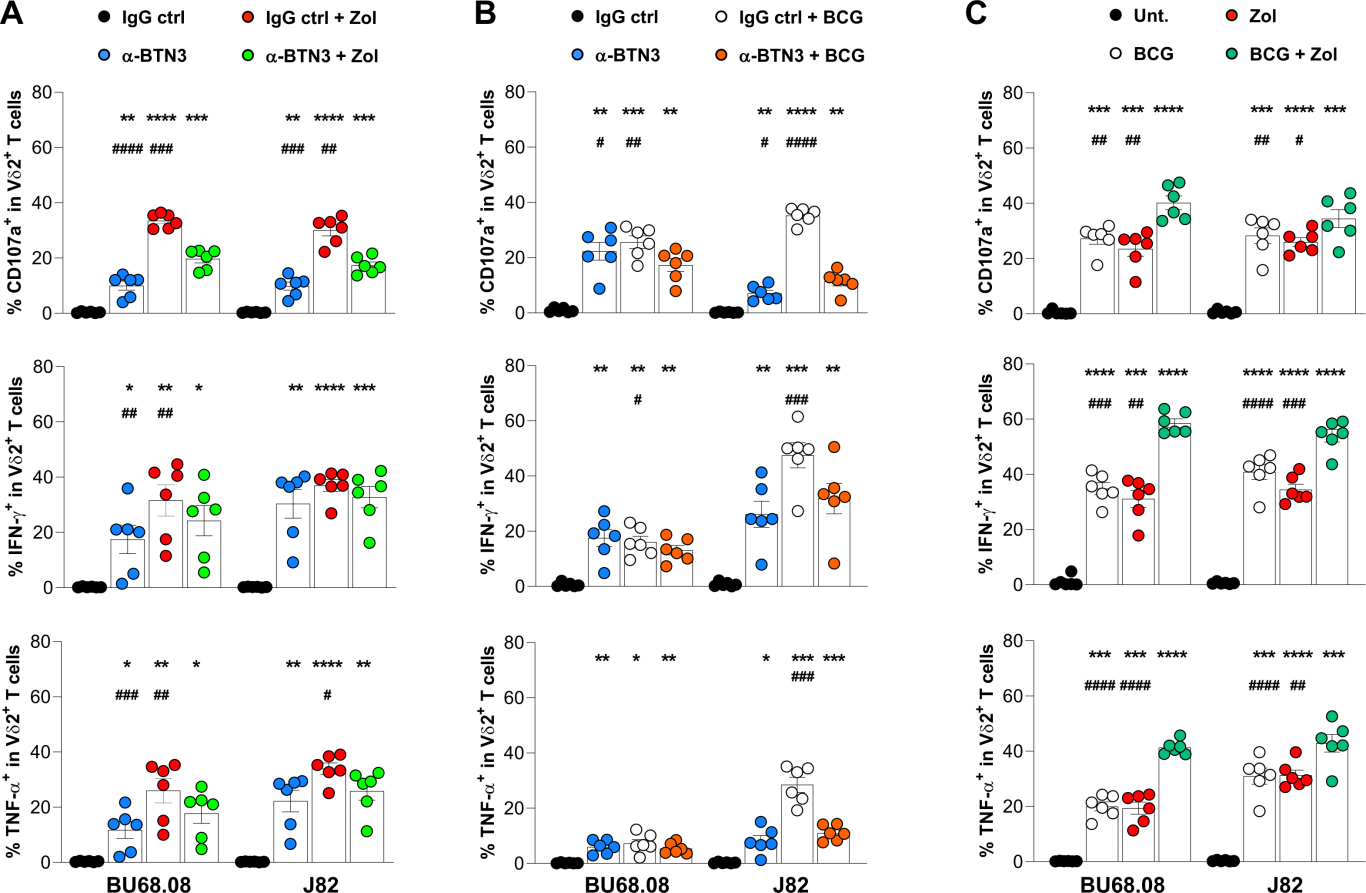
Vδ2 T-cell function against BCa cell lines on combinatorial treatments. Functional properties of Vδ2 T cells from HD PBMCs (n=6) on adjunction of agonistic α-BTN3 antibody on Zol-treated (A) or BCG-infected (B) Bu68.08 and J82 cell lines. (C) Evaluation of the synergistic effect of BCG infection and Zol treatment on the recognition of bladder tumor cell lines by Vδ2 T cells from HD PBMCs (n=6). Indicated p values were determined by one-way ANOVA followed by Dunnett’s post-test, comparing each condition with the untreated control (black stars) or with the combined treatment (black hash). * or # p<0.05, ** or ## p<0.01, *** or ### p<0.001, **** or #### p<0.0001. ANOVA, analysis of variance; BCa, bladder cancer; HD, healthy donors; PBMCs, peripheral blood monocytic cells.

Considering that the three tested treatments successfully activated Vδ2 T-cell antitumoral functions, we investigated whether combinational treatment could act synergistically. Therefore, Bu68.08 and J82 were treated with combined treatments, cocultured with PBMCs from HD, and Vδ2 T-cell functions were analyzed by flow cytometry. As shown in the figure 4A, B, no additive effect of Vδ2 T-cell cytotoxic capacity and cytokine production was observed when combining Zol and α-BTN3 or BCG and α-BTN3 treatments. The adjunction of the α-BTN3 antibody even seemed to decrease Zol and BCG effects (figure 4A, B). However, the combination of Zol and BCG treatment was superior in promoting both Vδ2 T-cell cytotoxic response and cytokine production than individual treatments (figure 4C).

### Combined Zol and BCG treatment promote the functionality of Vδ2 T cells from patients with BCa

**Figure 5:**
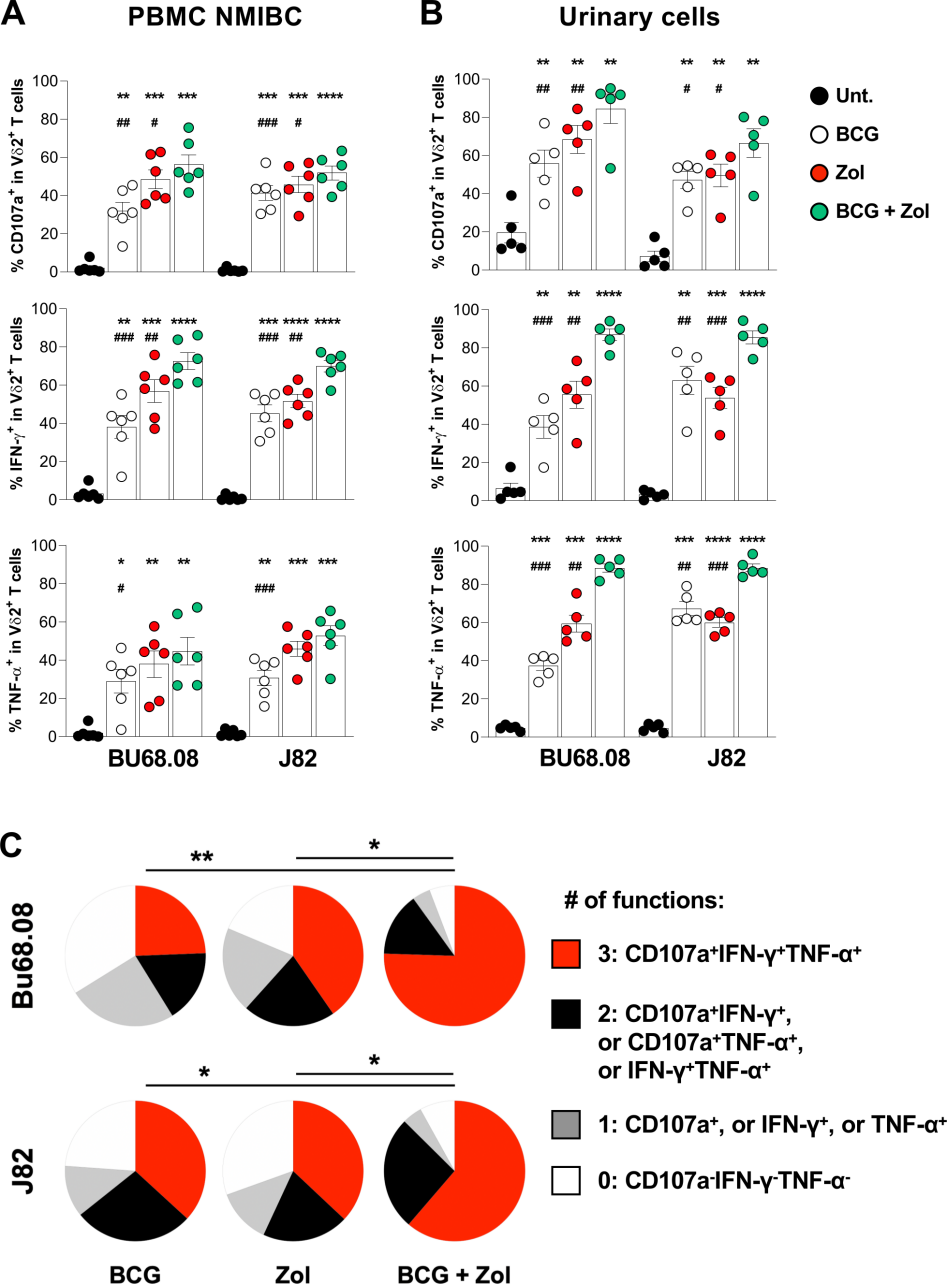
BCG and Zol combinatorial treatment boost circulating and urinary Vδ2 T-cell functions from NMIBC patients. Evaluation of cytotoxic capacity (CD107a) and cytokines (IFN-γ and TNF-α) production of (A) circulating (n=6) and (B) urinary (n=5) Vδ2 T cells from NMIBC patients on coculture with J82 and Bu68.08 cell lines treated with the combination of BCG and Zol. Indicated p values were determined by one-way ANOVA, comparing each condition with control (black stars) or combined treatment (black hash). (C) Determination of urinary Vδ2 T-cell polyfunctionality on coculture with BCa cell lines treated with BCG and Zol combined treatment. * or ^#^ p<0.05. ** or ^##^p<0.01. *** or ^###^p<0.001. **** or ^####^ p<0.0001. ANOVA, analysis of variance; NMIBC, non-muscle-invasive bladder cancer.

To validate the additive effect of BCG and Zol treatments in patients, PBMCs from patients with BCa were cocultured with BCa cell lines treated with combined treatment. For both BCa cell lines, we observed that circulating Vδ2 T cells from NMIBC patients exhibited improved cytotoxic function and cytokine production on combined therapy compared with single treatments (figure 5A), whereas the additive effect was only observed with the J82 cell line in MIBC patients (online supplemental figure 4C). In addition, to determine whether γδ T cells infiltrating the bladder may also respond to the combined treatment, freshly isolated urine T cells from NMIBC patients undergoing a BCG therapy were expanded in vitro^17^ and used in the functional assay. Surprisingly, expanded urinary T cells contain a relatively high frequency of γδ T cells, mainly composed of Vδ2 T cells (online supplemental figure 4D), suggesting that Vδ2 T cells may infiltrate the bladder during BCG therapy. Similar to previous results obtained with PBMCs, we observed a higher frequency of cytotoxic and cytokine-producing urinary Vδ2 T cells on combined treatment, compared with individual treatments (figure 5B). Finally, it is well known that T-cell exhaustion, including loss of polyfunctionality, is a hallmark of many cancers and chronic infections.^28^ Consequently, therapies restoring T-cell polyfunctionality were often associated with good clinical outcomes.^29 30^ As shown in figure 5C, polyfunctionality analysis showed that BCG and Zol combinatorial treatment enhances Vδ2 T-cell trifunctionality, as measured by the coexpression of CD107a, IFN-γ and TNF-α, compared with both individual treatments. Altogether, these data indicate that the Zol administration during BCG therapy might be beneficial to treat NMIBC patients by quantitatively and qualitatively boosting tumor-reactive Vδ2 T cells.

## Discussion

In the past decades, substantial evidence indicated that TILs are associated with a favorable prognosis in BCa.^4 5^ However, such observations were mainly based on conventional αβ T cells, while the role and the clinical relevance of unconventional γδ T cells in patients with BCa have not yet been fully assessed. In this present study, bulk transcriptomic analyses first revealed the positive impact of intratumor γδ T cells on the survival of locally advanced bladder tumor patients, similar to previous studies in other types of tumors.^27 31 32^ Vδ2 rather than Vδ1 T cells may play a significant role in bladder tumor control, since we observed that BTN3A1 expression, crucial ligand for Vδ2 T-cell activation, correlate with improved patients’ survival. Even though in silico prediction of infiltrating immune cells from bulk transcriptomic data is a powerful tool for dissecting the tumor microenvironment, such technic is hindered by overlapping signatures between cell subsets, which is particularly true for γδ T lymphocytes. Indeed, single-cell RNA sequencing dataset analysis from HDs PBMC showed that cells with γδ T-cell gene patterns are embedded in T and NK cell clusters.^33^ Therefore, gene-set signature-based methods to address γδ T-cell proportion should be taken with caution to avoid the misidentification of γδ T cells.

Subsequent flow cytometry analyses in peripheral blood samples revealed that NMIBC and MIBC patients with a high circulating Vδ2 T-cell frequency showed better survival. These findings raise the possibility that levels of blood Vδ2 T cells might be a useful prognostic biomarker for BCa, as recently shown in melanoma patients.^34^ In addition, both transcriptomic and flow cytometry analyses showed a lower proportion of γδ T cells in the tumor and the blood of patients with BCa, probably contributing to the compromised antitumor immune response in those patients. This highlights that immune escape mechanisms developed by the tumor may be harmful to γδ T cells.^35^

Furthermore, functional investigations unveiled that bladder tumor cells could be sensitized to Vδ2 T-cell recognition by using either amino bisphosphonate drugs (eg, Zoledronate) or agonistic α-BTN3 antibody.^11 12^ Even though PAgs derived from BCG extract are known to promote Vδ2 T-cell function,^14^ we further demonstrated that BCG-infected bladder tumor cell lines can activate cytolytic function and cytokine production of Vδ2 T cells. Of note, we found that viable BCG is required for optimal Vδ2 T-cell bladder tumor recognition on BCG infection. This indicates that active BCG internalization is essential for the interaction between PAgs from BCG (BCG-PAg) with the intracellular B30.2 domain of BTN3A1, thus promoting its active conformation^10 36^ and eventually Vδ2 T-cell activation. In line with this result, murine and clinical studies demonstrated that viable BCG is required for the activation and local recruitment of T cells, as well as BCG treatment efficacy.^37 38^ Moreover, combinational treatment functional assays revealed two interesting findings: (1) Agonistic α-BTN3 antibody exhibits opposite effects in the presence or absence of Zol. Such discrepancy was already described in Vγ9Vδ2-TCR reporter cells, and it has been postulated that agonistic α-BTN3 antibody may stabilize BTN3A1 into a suboptimal conformation able to slightly stimulate Vγ9Vδ2 T cells but prevent the PAg-induced BTN3A1 optimal conformation.^39 40^ Further studies are thus needed to ascertain this hypothesis and clarify the underlying molecular mechanisms. (2) In contrast, the combination of Zol and BCG treatment was better than the single treatment by increasing the proportion of tumor-reactive and the polyfunctionality of Vδ2 T cells. Since treated tumor cells were washed to remove any residual treatment and protein transport inhibitors were added at the initiation of the functional assay, we can hypothesize that such improvement is mediated through non-soluble factors. Interestingly, it has been shown that BCG-PAgs- and IPP-reactivity elicit distinct Vδ2 T-cell clonotypes.^41^ Although the detailed mechanism underlying such specificity remains unknown, the involvement of the Vγ9 CDR3 region seems critical for defining the Vγ9Vδ2 TCR reactivity.^42 43^ Therefore, Zol and BCG combinatorial treatment may trigger a larger and more diverse Vδ2 T-cell repertoire against bladder tumors. Further TCR diversity evaluation of tumor-reactive Vδ2 T cells on combinational treatment has to be performed to validate such hypothesis. Of note, few studies have described an infiltration of γδ T cells in the urine along the BCG therapy.^44 45^ We observed a high ratio of Vδ2/Vδ1 T cells in urinary expanded T cells from patient undergoing a BCG therapy. Such distribution probably reflects the differential proliferation rate on conventional in vitro expansion protocol (PHA, feeder cells, and IL-2). Further ex vivo characterization of urinary γδ T cells have to be performed to validate the contribution of each γδ T-cell subset during BCG therapy. Zol is an effective antiresorptive agent approved for preventing skeletal-related events in patients with cancer with bones metastases and is generally well tolerated. Zol treatment may also improve the survival rate of patients with BCa with bone metastases.^46^ In addition to its action on osteoclasts and Vδ2 T cells, Zol may show several direct and indirect antitumor effects, such as inhibition of tumor cell proliferation, induction of tumor cell apoptosis, reduction of angiogenesis, and polarization of macrophages toward M1 phenotype.^47^ Given that mycobacteria are potent inducers of Th1 responses required for BCG-mediated bladder tumor control, combining Zol with BCG may be a promising treatment in NMIBC patients that deserves further evaluation in Phase I/II clinical trial.

## Conclusions

Altogether, our data demonstrated the anti-bladder tumor function of Vδ2 T cells, which functional properties can be enhanced by combinational treatment of BCG and Zol, thus opening a new therapeutic approach for BCa.

## Data Availability

Data are available on reasonable request. All data are available on request to the corresponding author.

## References

[1] Sanli O, Dobruch J, Knowles MA, et al. Bladder cancer. Nat Rev Dis Primers 2017;3:17022. 10.1038/nrdp.2017.22 28406148

[2] Lamm DL. Efficacy and safety of Bacille Calmette-Guérin immunotherapy in superficial bladder cancer. Clin Infect Dis 2000;31 Suppl 3:S86–90. 10.1086/314064 11010830

[3] Witjes JA. Management of BCG failures in superficial bladder cancer: a review. Eur Urol 2006;49:790–7. 10.1016/j.eururo.2006.01.017 16464532

[4] Fridman WH, Pagès F, Sautès-Fridman C, et al. The immune contexture in human tumours: impact on clinical outcome. Nat Rev Cancer 2012;12:298–306. 10.1038/nrc3245 22419253

[5] Schneider AK, Chevalier MF, Derré L. The multifaceted immune regulation of bladder cancer. Nat Rev Urol 2019;16:613–30. 10.1038/s41585-019-0226-y 31501534

[6] Groh V, Steinle A, Bauer S, et al. Recognition of stress-induced MHC molecules by intestinal epithelial gammadelta T cells. Science 1998;279:1737–40. 10.1126/science.279.5357.1737 9497295

[7] Cordova A, Toia F, La Mendola C, et al. Characterization of human γδ T lymphocytes infiltrating primary malignant melanomas. PLoS One 2012;7:e49878. 10.1371/journal.pone.0049878 23189169 PMC3506540

[8] Corvaisier M, Moreau-Aubry A, Diez E, et al. V gamma 9V delta 2 T cell response to colon carcinoma cells. J Immunol 2005;175:5481–8. 10.4049/jimmunol.175.8.5481 16210656

[9] Willcox BE, Willcox CR. γδ TCR ligands: the quest to solve a 500-million-year-old mystery. Nat Immunol 2019;20:121–8. 10.1038/s41590-018-0304-y 30664765

[10] Harly C, Guillaume Y, Nedellec S, et al. Key implication of CD277/butyrophilin-3 (BTN3A) in cellular stress sensing by a major human γδ T-cell subset. Blood 2012;120:2269–79. 10.1182/blood-2012-05-430470 22767497 PMC3679641

[11] Compte E, Pontarotti P, Collette Y, et al. Frontline: characterization of BT3 molecules belonging to the B7 family expressed on immune cells. Eur J Immunol 2004;34:2089–99. 10.1002/eji.200425227 15259006

[12] Palakodeti A, Sandstrom A, Sundaresan L, et al. The molecular basis for modulation of human Vγ9Vδ2 T cell responses by CD277/butyrophilin-3 (BTN3A)-specific antibodies. J Biol Chem 2012;287:32780–90. 10.1074/jbc.M112.384354 22846996 PMC3463320

[13] Gober H-J, Kistowska M, Angman L, et al. Human T cell receptor gammadelta cells recognize endogenous mevalonate metabolites in tumor cells. J Exp Med 2003;197:163–8. 10.1084/jem.20021500 12538656 PMC2193814

[14] Constant P, Poquet Y, Peyrat MA, et al. The antituberculous Mycobacterium bovis BCG vaccine is an attenuated mycobacterial producer of phosphorylated nonpeptidic antigens for human gamma delta T cells. Infect Immun 1995;63:4628–33. 10.1128/iai.63.12.4628-4633.1995 7591116 PMC173665

[15] Yuasa T, Sato K, Ashihara E, et al. Intravesical administration of gammadelta T cells successfully prevents the growth of bladder cancer in the murine model. Cancer Immunol Immunother 2009;58:493–502. 10.1007/s00262-008-0571-9 18682944 PMC11029835

[16] Ji N, Mukherjee N, Shu Z-J, et al. γδ T Cells Support Antigen-Specific αβ T cell-Mediated Antitumor Responses during BCG Treatment for Bladder Cancer. Cancer Immunol Res 2021;9:1491–503. 10.1158/2326-6066.CIR-21-0285 34607803 PMC8691423

[17] Pieraerts C, Martin V, Jichlinski P, et al. Detection of functional antigen-specific T cells from urine of non-muscle invasive bladder cancer patients. Oncoimmunology 2012;1:694–8. 10.4161/onci.20526 22934261 PMC3429573

[18] Robertson AG, Kim J, Al-Ahmadie H, et al. Comprehensive molecular characterization of muscle-invasive bladder cancer. Cell 2017;171:540–56. 10.1016/j.cell.2017.09.007 28988769 PMC5687509

[19] Miao Y-R, Zhang Q, Lei Q, et al. ImmuCellAI: a unique method for comprehensive T-cell subsets abundance prediction and its application in cancer immunotherapy. Adv Sci 2020;7:1902880. 10.1002/advs.201902880 PMC7141005 32274301

[20] Zakeri N, Hall A, Swadling L, et al. Characterisation and induction of tissue-resident gamma delta T-cells to target hepatocellular carcinoma. Nat Commun 2022;13:1372. 10.1038/s41467-022-29012-1 35296658 PMC8927126

[21] Tang Z, Kang B, Li C, et al. GEPIA2: an enhanced web server for large-scale expression profiling and interactive analysis. Nucleic Acids Res 2019;47:W556–60. 10.1093/nar/gkz430 31114875 PMC6602440

[22] Pizzolato G, Kaminski H, Tosolini M, et al. Single-cell RNA sequencing unveils the shared and the distinct cytotoxic hallmarks of human TCRVδ1 and TCRVδ2 γδ T lymphocytes. Proc Natl Acad Sci U S A 2019;116:11906–15. 10.1073/pnas.1818488116 31118283 PMC6576116

[23] Chevalier MF, Trabanelli S, Racle J, et al. ILC2-modulated T cell-to-MDSC balance is associated with bladder cancer recurrence. J Clin Invest 2017;127:2916–29. 10.1172/JCI89717 28650339 PMC5531411

[24] O'Toole C, Price ZH, Ohnuki Y, et al. Ultrastructure, karyology and immunology of a cell line originated from a human transitional-cell carcinoma. Br J Cancer 1978;38:64–76. 10.1038/bjc.1978.164 687519 PMC2009694

[25] López-Ratón M, Rodríguez-Álvarez MX, Suárez CC, et al. OptimalCutpoints : An R Package for Selecting Optimal Cutpoints in Diagnostic Tests. J Stat Softw 2014;61:1–36. 10.18637/jss.v061.i08

[26] Yu D, Hwang W-T. Optimal cutoffs for continuous biomarkers for survival data under competing risks. Commun Stat Simul Comput 2019;48:1330–45. 10.1080/03610918.2017.1410713

[27] Tosolini M, Pont F, Poupot M. Assessment of tumor-infiltrating TCRVgamma9Vdelta2 gammadelta lymphocyte abundance by deconvolution of human cancers microarrays. Oncoimmunology 2017;6:e1284723. 28405516 10.1080/2162402X.2017.1284723 PMC5384348

[28] Wherry EJ. T cell exhaustion. Nat Immunol 2011;12:492–9. 10.1038/ni.2035 21739672

[29] Tanyi JL, Chiang CL-L, Chiffelle J, et al. Personalized cancer vaccine strategy elicits polyfunctional T cells and demonstrates clinical benefits in ovarian cancer. NPJ Vaccines 2021;6:36. 10.1038/s41541-021-00297-5 33723260 PMC7960755

[30] Yuan J, Gnjatic S, Li H, et al. CTLA-4 blockade enhances polyfunctional NY-ESO-1 specific T cell responses in metastatic melanoma patients with clinical benefit. Proc Natl Acad Sci U S A 2008;105:20410–5. 10.1073/pnas.0810114105 19074257 PMC2629307

[31] Zhao N, Dang H, Ma L, et al. Intratumoral γδ T-cell infiltrates, chemokine (C-C motif) ligand 4/Chemokine (C-C motif) ligand 5 protein expression and survival in patients with hepatocellular carcinoma. Hepatology 2021;73:1045–60. 10.1002/hep.31412 32502310 PMC9175512

[32] Girard P, Charles J, Cluzel C, et al. The features of circulating and tumor-infiltrating γδ T cells in melanoma patients display critical perturbations with prognostic impact on clinical outcome. Oncoimmunology 2019;8:1601483. 10.1080/2162402X.2019.1601483 31413911 PMC6682366

[33] Lo Presti E, Dieli F, Fourniè JJ, et al. Deciphering human γδ T cell response in cancer: lessons from tumor-infiltrating γδ T cells. Immunol Rev 2020;298:153–64. 10.1111/imr.12904 32691450

[34] Wistuba-Hamprecht K, Martens A, Haehnel K, et al. Proportions of blood-borne Vδ1+ and Vδ2+ T-cells are associated with overall survival of melanoma patients treated with ipilimumab. Eur J Cancer 2016;64:116–26. 10.1016/j.ejca.2016.06.001 27400322 PMC5201188

[35] Wesch D, Kabelitz D, Oberg H-H. Tumor resistance mechanisms and their consequences on γδ T cell activation. Immunol Rev 2020;298:84–98. 10.1111/imr.12925 33048357

[36] Espinosa E, Belmant C, Sicard H, et al. Y2K+1 state-of-the-art on non-peptide phosphoantigens, a novel category of immunostimulatory molecules. Microbes Infect 2001;3:645–54. 10.1016/S1286-4579(01)01420-4 11445451

[37] Redelman-Sidi G, Glickman MS, Bochner BH. The mechanism of action of BCG therapy for bladder cancer--a current perspective. Nat Rev Urol 2014;11:153–62. 10.1038/nrurol.2014.15 24492433

[38] Martino A, Casetti R, Sacchi A, et al. Central memory Vgamma9Vdelta2 T lymphocytes primed and expanded by Bacillus Calmette-Guérin-infected dendritic cells kill mycobacterial-infected monocytes. J Immunol 2007;179:3057–64. 10.4049/jimmunol.179.5.3057 17709520

[39] Starick L, Riano F, Karunakaran MM, et al. Butyrophilin 3A (BTN3A, CD277)-specific antibody 20.1 differentially activates Vγ9Vδ2 TCR clonotypes and interferes with phosphoantigen activation. Eur J Immunol 2017;47:982–92. 10.1002/eji.201646818 28386905

[40] Franchini D-M, Michelas M, Lanvin O, et al. BTN3A1-antibodies and phosphoantigens: TCRVγ9Vδ2 "see" the difference. Eur J Immunol 2017;47:954–7. 10.1002/eji.201747058 28597565

[41] Spencer CT, Abate G, Blazevic A, et al. Only a subset of phosphoantigen-responsive gamma9delta2 T cells mediate protective tuberculosis immunity. J Immunol 2008;181:4471–84. 10.4049/jimmunol.181.7.4471 18802050 PMC2670066

[42] Bukowski JF, Morita CT, Band H, et al. Crucial role of TCR gamma chain junctional region in prenyl pyrophosphate antigen recognition by gamma delta T cells. J Immunol 1998;161:286–93. 9647235

[43] Evans PS, Enders PJ, Yin C, et al. In vitro stimulation with a non-peptidic alkylphosphate expands cells expressing Vgamma2-Jgamma1.2/Vdelta2 T-cell receptors. Immunology 2001;104:19–27. 10.1046/j.1365-2567.2001.01282.x 11576216 PMC1783282

[44] Alexandroff A, Black J, Bollina P, et al. Differential production of gamma delta T cells in the urine of bladder cancer patients receiving Bacillus Calmette Guerin immunotherapy. Int J Oncol 1997;10:387–93. 10.3892/ijo.10.2.387 21533389

[45] Ponticiello A, Perna F, Maione S, et al. Analysis of local T lymphocyte subsets upon stimulation with intravesical BCG: a model to study tuberculosis immunity. Respir Med 2004;98:509–14. 10.1016/j.rmed.2003.12.003 15191035

[46] Zaghloul MS, Boutrus R, El-Hossieny H, et al. A prospective, randomized, placebo-controlled trial of zoledronic acid in bony metastatic bladder cancer. Int J Clin Oncol 2010;15:382–9. 10.1007/s10147-010-0074-5 20354750

[47] Van Acker HH, Anguille S, Willemen Y, et al. Bisphosphonates for cancer treatment: mechanisms of action and lessons from clinical trials. Pharmacol Ther 2016;158:24–40. 10.1016/j.pharmthera.2015.11.008 26617219

