# Supplementary material for: Vδ2 T cells are associated with favorable clinical outcomes in patients with bladder cancer and their tumor reactivity can be boosted by BCG and zoledronate treatments
Source: J Immunother Cancer. 2022 Aug 24;10(8):e004880. doi: 10.1136/jitc-2022-004880 (PMC9413168; doi:10.1136/jitc-2022-004880)
Supplement: Supplementary data [file jitc-2022-004880supp002.pdf]

Supplementary Table 1 – Characteristics of non-muscle-invasive bladder cancer patients

Characteristics	All patients	Low Vδ2 T cells	High Vδ2 T cells	P value
No. of patients	40	19	21	
Age (yr), median (IQR)	71 (64.25-76.75)	74 (66-83)	69 (62-74)	ns (P=0.071)[a]
**Sex, *n***				
Male	29 (72.5)	13 (68.4)	16 (76.2)	ns (P=0.73)[b]
Female	11 (27.5)	6 (31.6)	5 (23.8)	
**Tumor status, *n* (%)**				ns (P=0.49)[b#]
pTa	20 (50)	8 (42.1)	12 (57.1)	
pT1	14 (35)	8 (42.1)	6 (28.6)	
pTIS	6 (15)	3 (15.8)	3 (14.3)	
Any CIS positive	9 (22.5)	5 (26.3)	4 (19.05)	
**Grade, *n* (%)**				
Low grade	19 (47.5)	6 (31.6)	13 (61.9)	ns (p=0.07)[b]
High grade	21 (52.5)	13 (68.4)	8 (38.1)	
**No. of tumors, *n* (%)**				
1	32 (80)	14 (73.7)	16 (76.2)	ns (P=0.99)[b]
≥ 2	8 (20)	5 (26.3)	5 (23.8)	

[a]Unpaired Student's t test; [b]Fisher's exact test; [c]$\chi 2$ test; [#]comparison between pTa and pT1, CIS = carcinoma in situ; IQR = interquartile range.

Supplementary Table 2 – Characteristics of muscle-invasive bladder cancer patients

Characteristics	All patients	Low Vδ2 T cells	High Vδ2 T cells	P value
No. of patients	40	16	24	
Age (yr), median (IQR)	72.5 (65.25-78)	70 (65.25-75)	74 (65.25-80)	ns (P=0.29)[a]
**Sex, *n***				
Male	33 (82.5)	14 (87.5)	19 (79.2)	ns (P=0.68)[b]
Female	7 (17.5)	2 (12.5)	5 (20.8)	
**Tumor status, *n* (%)**				ns (P=0.23)[c]
pT2	15 (37.5)	5 (31.25)	10 (41.7)	
pT3	19 (47.5)	10 (62.5)	9 (37.5)	
pT4	6 (15)	1 (6.25)	5 (20.8)	
**Draining lymph node status, *n* (%)**				
Nx	6 (15)	1 (6.25)	5 (20.8)	
N0	22 (55)	7 (43.75)	15 (62.5)	ns (P=0.16)[b]
> N1	12 (30)	8 (50)	4 (16.7)	
**Concomitant prostate cancer, *n* (%)**	18 (45)	8 (50)	10 (41.6)	
**Chemotherapy, *n* (%)**	19 (47.5)	8 (50)	11 (45.8)	

[a]Unpaired Student's t test; [b]Fisher's exact test; [c]$\chi 2$ test; IQR = interquartile range.

Supplementary Table 3 – Characteristics of bladder cancer patients undergoing BCG therapy

Characteristics	All patients
No. of patients	25
Age (yr), median (IQR)	67 (62.5-74.5)
**Sex, *n***	
Male	24 (96)
Female	1 (4)
**Tumor status, *n* (%)**	
pTa	5 (20)
pT1	12 (48)
pT2	1 (4)[§]
pTIS	7 (28)
Any CIS positive	14 (56)
**Grade, *n* (%)**	
Low grade	0 (0)
High grade	25 (100)
**No. of tumors, *n* (%)**	
1	16 (64)
≥2	9 (36)

[§]This patient was diagnosed with MIBC but refused cystectomy and instead received BCG therapy,
CIS = carcinoma in situ; IQR = interquartile range.
