# Supplementary material for: Vδ2 T cells are associated with favorable clinical outcomes in patients with bladder cancer and their tumor reactivity can be boosted by BCG and zoledronate treatments
Source: J Immunother Cancer. 2022 Aug 24;10(8):e004880. doi: 10.1136/jitc-2022-004880 (PMC9413168; doi:10.1136/jitc-2022-004880)
Supplement: Supplementary data [file jitc-2022-004880supp001.pdf]

Supplementary Figure 1

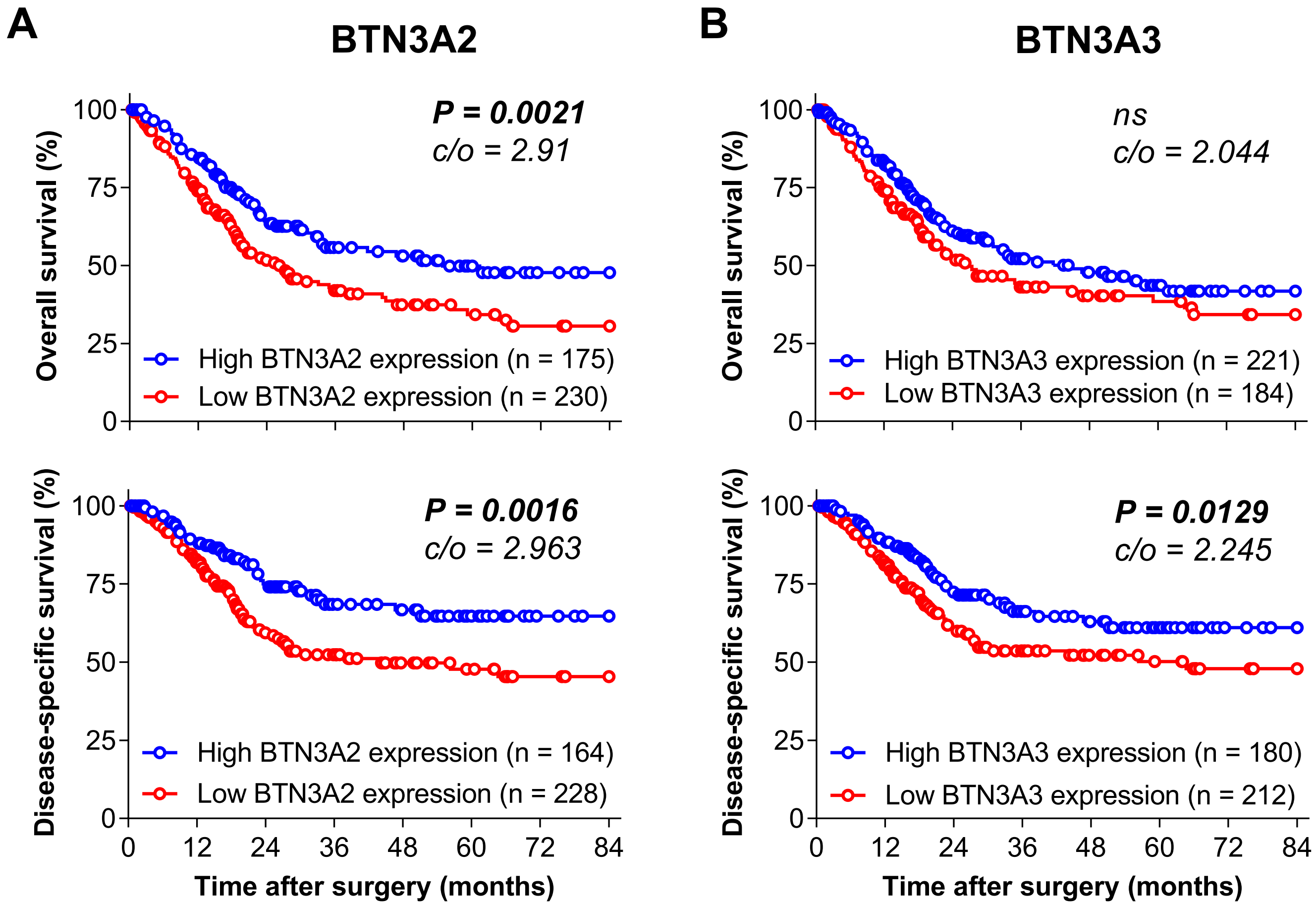

Supplementary figure 1. BTN3A2 and BTN3A3 from TCGA analysis
Overall (n = 405) and disease-specific (n = 392) survival analysis based on BTN3A2 **(A)** and BTN3A3 **(B)** mRNA expression obtained from TCGA cohort. Cutoff values (c/o) are indicated.

Supplementary Figure 2

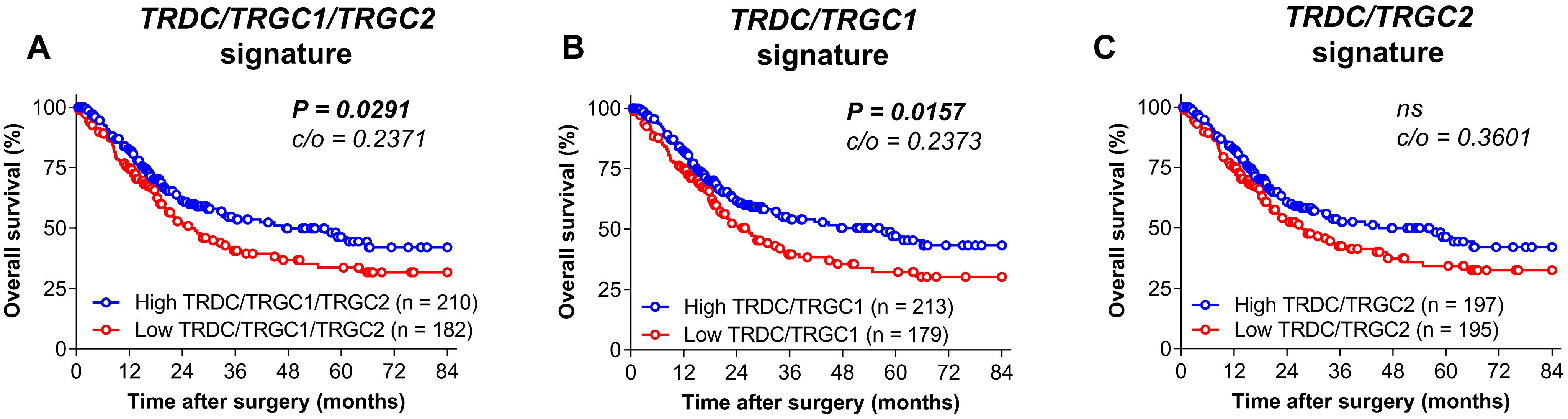

Supplementary figure 2. Survival analysis of γδ TCR gene signatures (*TRDC*, *TRGC1* and *TRGC2*) from TCGA dataset. **(A)** γδ (*TRDC*/*TRGC1*/*TRGC2* signature), **(B)** Vδ2 (*TRDC*/*TRGC1* signature) and **(C)** non-Vδ2 (*TRDC*/*TRGC2* signature) T-cell gene score proportions in bladder tumor tissue from the TCGA cohort (n = 392) according to their survival status. Cutoff values (c/o) are indicated.

Supplementary Figure 3

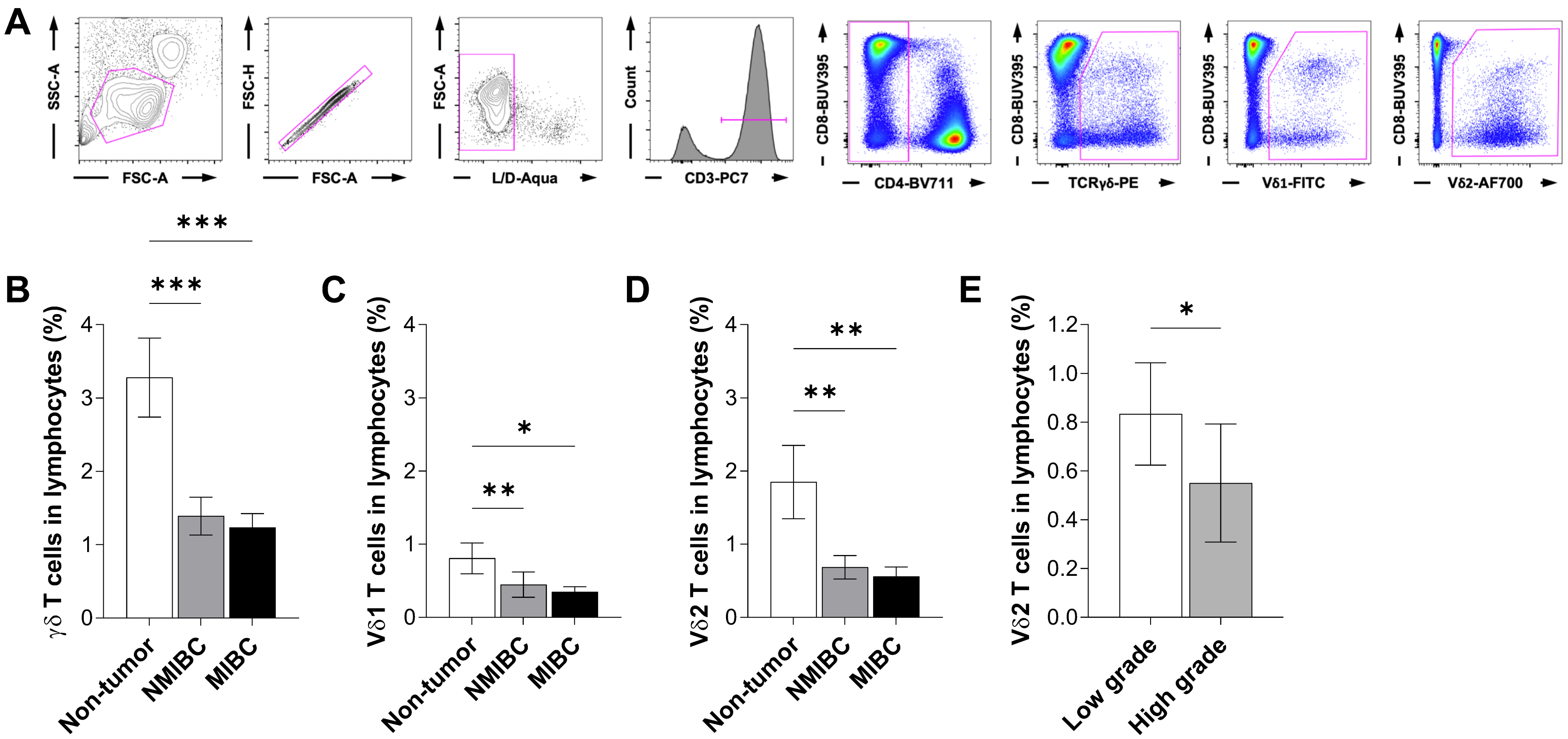

Supplementary figure 3. Evaluation of circulating γδ T-cell frequency in NMIBC and MIBC patients
(A) Representative example of gating strategy. Lymphocytes were gated on size and scatter, followed by doublet and dead cell exclusion before subgating into total γδ ($CD3^+CD4^-panTCR\gamma\delta^+$), Vδ1 ($CD3^+CD4^-TCRV\delta1^+$) and Vδ2 ($CD3^+CD4^-TCRV\delta2^+$) T cells. Frequency of circulating γδ **(B)**, Vδ1 **(C)** and Vδ2 **(D)** T cells in non-tumor (n = 20), NMIBC (n = 40) and MIBC patients (n = 40). **(E)** Frequency of blood Vδ2 T cells in from NMIBC patients with low- or high-grade tumor lesions. * $P < 0.05$. ** $P < 0.01$. *** $P < 0.001$.

Supplementary Figure 4

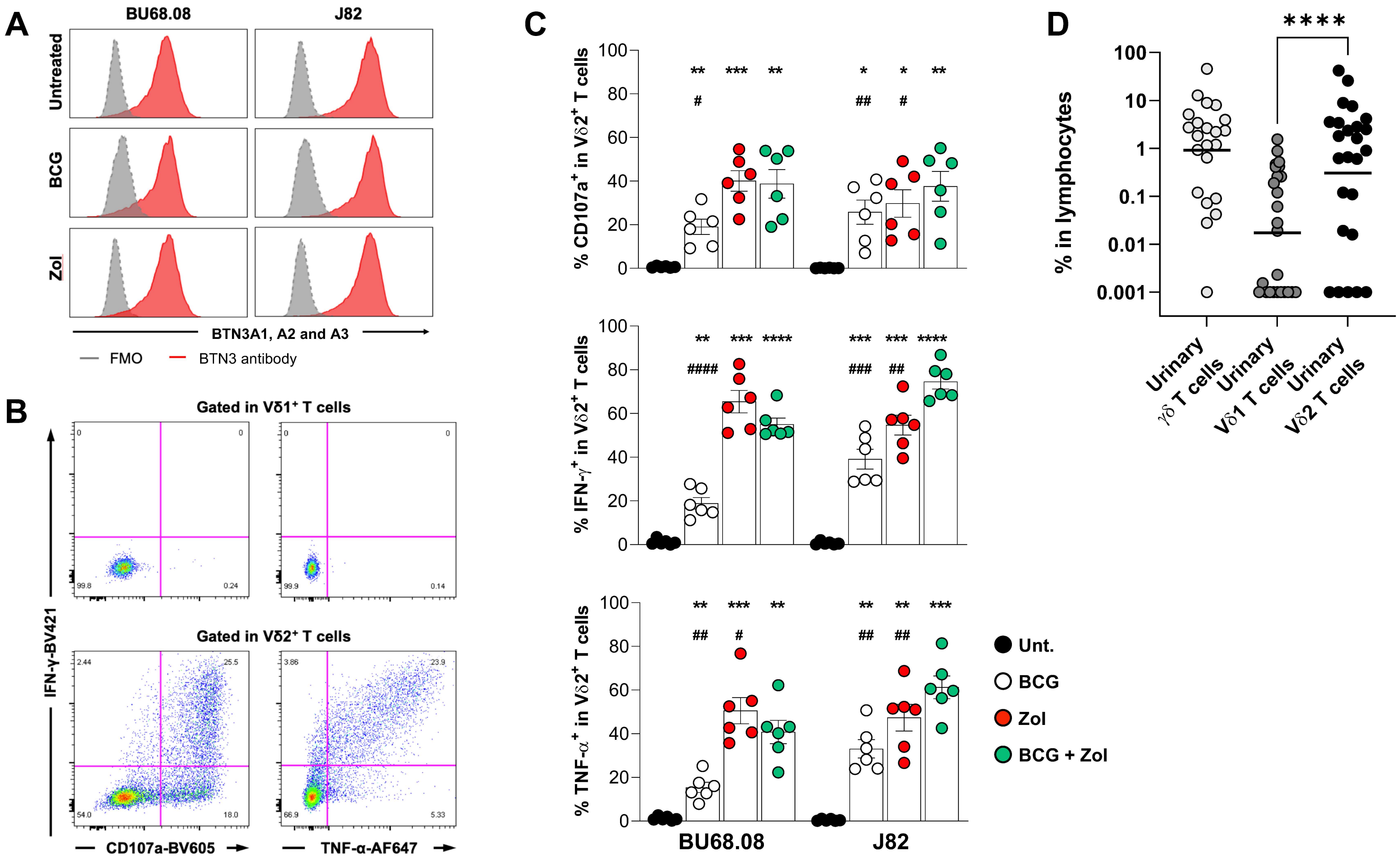

Supplementary figure 4. Functional properties of circulating γδ T cells from HD and MIBC patients and frequency of γδ T cells in urinary T cell lines

(A) Representative FACS expression of BTN3 on BU68.08 and J82 bladder tumor cell lines treated or not with BCG or Zol (FMO: Fluorescence Minus One). **(B)** Representative FACS dot plot of CD107a, IFN-γ and TNF-α expression in Vδ1 and Vδ2 T cells from PBMCs of HD upon co-culture with BCG-infected Bu68.08 (MOI = 10). **(C)** Evaluation of functional properties of circulating Vδ2 T cells from MIBC patients (n = 6) upon coculture with J82 and Bu68.08 cell lines treated with the combination of BCG and Zol. Indicated *P* values were determined by one-way ANOVA, comparing each condition with control (black stars) or combined treatment (black hash). **(D)** Frequency of total γδ (n=22), Vδ1 and Vδ2 T cells (n=25) in urinary T cell lines from NMIBC patients undergoing BCG therapy. Wilcoxon test was performed to assess the statistical difference between Vδ1 and Vδ2 T-cells frequency. * or $^{\#}$ $P < 0.05$. ** or $^{\#\#}$ $P < 0.01$. *** or $^{\#\#\#}$ $P < 0.001$. **** or $^{\#\#\#\#}$ $P < 0.0001$.
